# Accelerated Identification of Proteins by Mass Spectrometry by Employing Covalent Pre-Gel Staining with Uniblue A

**DOI:** 10.1371/journal.pone.0031438

**Published:** 2012-02-17

**Authors:** Marco A. Mata-Gómez, Matthew T. Yasui, Armando Guerrero-Rangel, Silvia Valdés-Rodríguez, Robert Winkler

**Affiliations:** 1 Department of Biotechnology and Food Engingeering, Tecnológico de Monterrey, Monterrey, Nuevo León, Mexico; 2 School of Chemical, Biological and Environmental Engineering, Oregon State University, Corvallis, Oregon, United States of America; 3 Department of Biotechnology and Biochemistry, CINVESTAV Unidad Irapuato, Irapuato, Guanajuato, Mexico; Aligarh Muslim University, India

## Abstract

**Background:**

The identification of proteins by mass spectrometry is a standard method in biopharmaceutical quality control and biochemical research. Prior to identification by mass spectrometry, proteins are usually pre-separated by electrophoresis. However, current protein staining and de-staining protocols are tedious and time consuming, and therefore prolong the sample preparation time for mass spectrometry.

**Methodology and Principal Findings:**

We developed a 1-minute covalent pre-gel staining protocol for proteins, which does not require de-staining before the mass spectrometry analysis. We investigated the electrophoretic properties of derivatized proteins and peptides and studied their behavior in mass spectrometry. Further, we elucidated the preferred reaction of proteins with Uniblue A and demonstrate the integration of the peptide derivatization into typical informatics tools.

**Conclusions and Significance:**

The Uniblue A staining method drastically speeds up the sample preparation for the mass spectrometry based identification of proteins. The application of this chemo-proteomic strategy will be advantageous for routine quality control of proteins and for time-critical tasks in protein analysis.

## Introduction

Proofing the identity of compounds during the manufacturing chain is a basic obligation in the pharmaceutical industries. Adequate quality control procedures are therefore mandatory and strictly supervised by regulatory bodies [1]. Additionally, fake drugs threaten the health of patients [2]–[4]. This makes additional quality controls necessary for products, which are already in circulation or imported. But also in biological research and development, the confirmation of the identity of molecules is crucial. This is especially true for laboratories working with proteins, since those are usually purified from complex mixtures and difficult to distinguish by their biochemical properties only.

Nowadays, proteins in most of the cases are identified based on mass spectrometry (MS) data, since current MS methods offer high sensitivity, speed and accuracy and hence permit reliable conclusions on the nature of a protein in reasonable time. Moreover, MS methods are applicable to virtually any protein and are not limited to the N-terminal sequence such as Edman sequencing.

Usually proteins need to be pre-separated, before they can be subjected to MS analysis. This is efficiently done by gel electrophoresis, which has the additional advantage to remove low molecular weight contaminants such as salts. For extremely complex samples, such as entire cell disintegrates with several thousand proteins, a two-dimensional gel electrophoresis (2D-GE) is necessary, which separates the proteins first by their isoelectric point and subsequently by their molecular weight [5]. However, 2D-GE is time and labor intense.

For analyzing partially purified or pure proteins, a one-dimensional gel electrophoresis (1D-GE) [6] is sufficient and provides the advantage, that several samples can be run in parallel on a single gel. Considering the much higher possible through-put compared to two-dimensional gel electrophoresis, we focus in our study on one-dimensional gel electrophoreses.

Independently, if 1D-GE or 2D-GE is chosen, the proteins need to be stained or labeled in order to be visible. In some cases, a selective stain might be applicable [7]. But for most cases, a general protein dye needs to be applied. The most sensitive protein stain, which is visible at natural light, is the silver staining. However, it is cumbersome and troubling in mass spectrometry analyses [8]. Therefore, the less sensitive Coomassie stain became the current de facto standard for protein staining [9]. Several protocols for Coomassie staining are given in the literature, which are either optimized for sensitivity, speed or mass spectrometry compatibility [10], [11]. Out of those, the staining with colloidal Coomassie is currently the method of choice, if the samples are intended for later analysis by mass spectrometry. However, considering the quickest protocols, three hours are necessary for colloidal Coomassie staining [11], and another four hours for preparing selected gel pieces for MS [12]–[15]. A significant part of this time is consumed by de-staining steps.

In comparison, the 1D-GE and the MS analysis take only about one to two hours each.

Besides the time issue, many tedious manual steps are necessary for the processing of Coomassie stained gel pieces, which increases the risk of sample contamination, for example by human keratin. Automation of the sample processing is possible, but its costs are considerable and the reliability and flexibility of robots is sometimes not satisfactory.

Altogether, we identified a vast potential for optimization in the sample preparation for mass spectrometry; especially in the protein staining/de-staining procedures. We sought after a protein staining method, which reduces the sample preparation effort before mass spectrometry to a minimum and consequently permits faster protein identification results. The requirements for such a staining method would be: Rapidity, visibility of stained proteins at natural light, compatibility with gel electrophoresis, compatibility with mass spectrometry and current data processing work-flows as well as simple adoption to existing laboratory procedures.

In the following study we demonstrate how those conditions can be met by covalent pre-gel staining of the proteins with Uniblue A.

## Results

### Covalent staining procedure and electrophoretic properties of derivatized proteins

**Figure 1:**
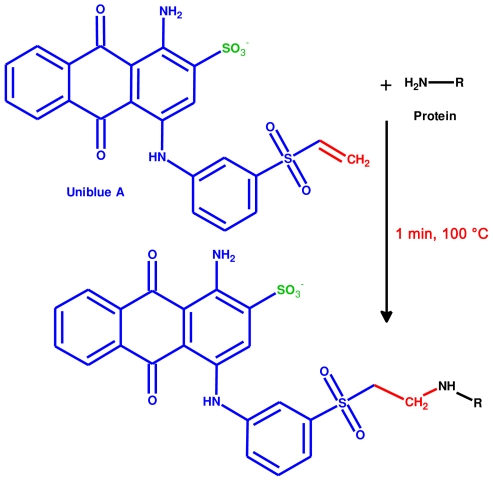
Covalent staining of proteins by nucleophilic addition of Uniblue A. The vinyl sulfone group (red) reacts with primary amines, preferably on lysine residues. The sulfate group (green) supports the solubility of the dye and affects the ionization properties of the labeled peptide during mass spectrometry measurements.

**Figure 2:**
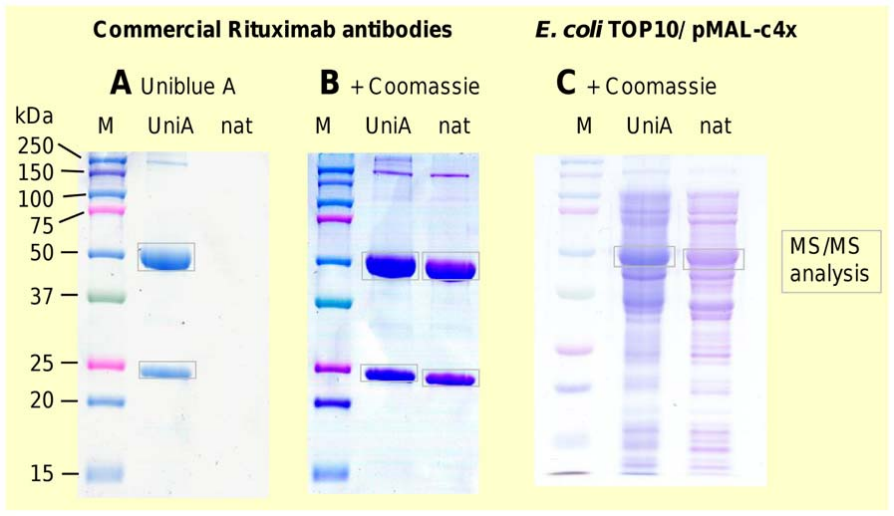
Electrophoretic properties of Uniblue A stained proteins. A) SDS-PAGE gel showing pre-stained molecular weight marker and Uniblue A (Uni A) derivatized Rituximab antibody chains. The staining was achieved within 1 minute. The third lane contains the equal concentration of un-derivatized Rituximab sample (nat). B) The same gel after subsequent staining with Coomassie, now also revealing the un-derivatized Rituximab sample. Staining intensity and protein profiles are comparable. C) *E. coli* TOP10, transformed with pMAL-c4x and auto-induced. Uniblue A (Uni A) derivatized and un-derivatized (nat) disintegration sample display comparable protein profiles after Coomassie staining. The assumed recombinant protein band was cut and subjected to nanoLC-MS/MS analysis, confirming the identity with >80% MS/MS based sequence coverage in both samples.

After some theoretical considerations and initial testing of several reactive protein dyes, Uniblue A seemed to be the most promising candidate, due to its solubility in water, commercial availability with adequate purity and low price. Additionally, its blue color aids in achieving a sufficient optical contrast. Uniblue A exhibits broad and intense absorption in the visible range with a maximum at 593.5 nm (Supplemental Fig. S1, λ_max_ = 596 nm according to Sigma Aldrich). This is practically the same absorption maximum as for Coomassie with a λ_max_ of 595 nm [16]. Therefore the same settings for the scanning of gels can be used for obtaining the best contrast. Since we suspected a reaction with amines (Fig. 1) at basic pH, we performed the staining reaction in an amine-free NaHCO_3_ buffer at a pH of 8–9. We discovered that staining can be obtained at different temperatures, ranging from 37°C up to 100°C. But whereas the reaction requires about 1 hour at 60°C, sufficient covalent pre-gel staining of the protein with Uniblue A can be obtained within only one minute at 100°C (Fig. 2A). Prolonged incubation at this temperature results in thermal protein degradation.

Further steps of the protocol include quenching of excess Uniblue A, reduction and alkylation. Altogether, the sample preparation for the SDS-PAGE can be completed in less than ten minutes. Additionally to the blue protein bands, also the reaction products with Tris buffer are visible and serve as running front indicator. Those low-molecular compounds disappear rapidly during the fixing of the gel.

For recombinant cystatin we determined a quantitative sensitivity of about 1 µg protein. This is less sensitive than current Coomassie staining protocols. However, the Uniblue A derivatization is fully compatible with subsequent Coomassie staining. Therefore, the intensity of protein bands gels can be increased by double-staining, if required (Fig. 2B, C and S3). Clearly, several of the advantages of the Uniblue A protocol would be lost after sequential staining, in particular the saving of analysis time. However, even assuming insufficient staining by Uniblue A for some samples, only a few minutes are required for the sample preparation, which is very little in comparison to several hours, which generally can be saved. As an analytical strategy, several lanes of the same sample, with and without prior Uniblue A derivatization, can be run in the same gel. In this approach, one lane with Uniblue A derivatized proteins could be used as internal standard for the progress of electrophoresis and for rapid identification by MS, whereas the other lanes of derivatized or un-derivatized protein could be subsequently stained, in order to evaluate purity.

The apparent molecular weights of pre-stained and un-labeled Coomassie stained proteins are in agreement (see Fig. 2B, C and S2). Hence, the electrophoretic mobility of the proteins is not changed significantly by their covalent staining, which is in congruence with previous studies employing dabsyl chloride [17] or Remazol dyes [18]. Presumably, these small appendices do not contribute to the binding of SDS.

On the other side, the negatively charged Uniblue A does strongly influences the isoelectric point of the derivatized proteins. Modified proteins are shifted towards the basic region of a 2D gel. Even prolonged isoelectric focusing does not result in defined spots (Fig. S4). The 2D analysis also reveals that increased derivatization leads to more diffuse spots, although the apparent molecular weight is not affected significantly.

Sensitivity and resolution are reduced for pre-stained proteins, but protein patterns of pre-stained and un-labeled Coomassie stained proteins are comparable, as demonstrated for the *Escherichia coli* disintegrate (Fig. 2C).

For SDS-PAGE gels intended for subsequent mass spectrometric analyses, the staining intensity and the resolution are perfectly adequate.

### Shortened work-up for mass spectrometry and peptide tracking

De-staining is not required for the work-up of gel pieces. Also reduction and alkylation can be skipped, since those steps are already integrated into the SDS-PAGE sample preparation. In comparison to the current best-in-class methods, the staining time could be reduced from three hours to less than ten minutes, and the sample work-up time from four hours to about two hours. In total, the required sample processing time was condensed to less than a third, and the manual handling steps could be significantly reduced, which reduces the risk of contamination. No stain particles are present, which reduces the chance of blockages which occasionally occur in the NanoLC analysis of Coomassie stained samples.

Tagged proteins and peptides display color in the visible spectrum and their fate can be tracked visually. This allows for the direct monitoring of sample processing steps, such as extraction and re-dissolution of peptides. This feature facilitates optimization and validation of sample preparation methods.

The additional sulfate group increases the solubility of derivatized proteins and peptides, which supports their extraction, especially in cases of very hydrophobic species.

### Elucidation of amino acid serving as reaction partner for Uniblue A

Uniblue A contains a single vinyl sulfone group that may react with primary amines via nucleophilic addition (Fig. 1). Covalently modified residues will have a defined monoisotopic mass shift of 484.0399 Da.

However, also other potential reaction partners such as sulfhydryl groups or hydroxyl groups might be possible. The actual reaction needed to be evaluated by mass spectrometry data. Therefore the data sets were run allowing for potential Uniblue A modifications on lysine, cysteine, asparagine, glutamine, threonine, arginine, and tyrosine. Surprisingly, it turned out, that under the given conditions only lysine residues were derivatized, but neither other amine containing residues such as asparagine and glutamine nor alternative functional groups. Therefore it can be suggested that the ε-amino group of lysine is the preferred reaction partner for a nucleophilic addition of Uniblue A. Moreover, only a fraction of the lysines was derivatized. Based on assigned peptide spectra, up to 17 Uniblue A modifications were found for BSA, a lysine rich protein (Tab. 1). Surprisingly, only few or no Uniblue A modifications were found for other samples, although the protein was successfully stained, as evaluated visually from the SDS-PAGE gels. This indicates that the sensitivity is reduced for Uniblue A derivatized peptides. But it also has to be kept in mind, that this quantification method for judging the ratio of modified peptides, the so-called “spectral counting”, is limited and might show huge variations, especially when it comes to low abundance peptides [19]. On the other side, when detected, Uniblue A modified peptides exhibit a different, chemically assisted, fragmentation behavior, which supports their evaluation with high significance, as discussed below.

These data are crucial for the design of efficient database searches, since only one potential modification site, namely +484.0399 Da at lysine, has to be considered.

The successful staining of cysteine-free recombinant cystatin adds biochemical proof that the staining does not depend on the presence of cysteine (Fig. S2, S3, S4).

### Automated and manual MS/MS data evaluation and integration of modification into standard bioinformatic work-flows

**Figure 3:**
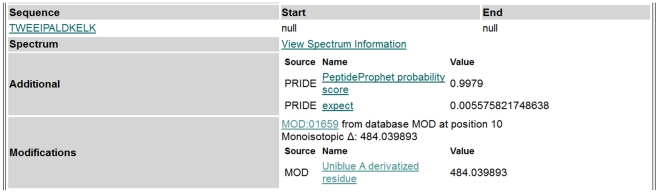
Annotation of a Uniblue A derivatization in the PRIDE repository. The peptide TWEEIPALDKELK was modified at position 10 (MOD:01659) and belongs to the identified recombinant protein MALELZ|MALELZ_LACZA|MALE-lacZ of experiment # 13516.

Raw MS/MS data were converted into mzXML and evaluated automatically (see Materials and Methods). In short, the search was performed against a concatenated target-decoy database [20] using the Open Mass Spectrometry Search Algorithm [21] (OMSSA). The peptide hits were validated by PeptideProphet [22] and ProteinProphet [23]. After this automatic processing, the raw data and identification results could be easily converted into valid PRoteomics IDEntifications database [24] (PRIDE) XML, using the PRIDE converter tool [25], and uploaded to the repository. Covalent derivatization with Uniblue A has been added by the PRIDE team as a protein modification (PSI-MOD) for the ontology lookup service (OLS) with the comma separated value (CSV) term MOD: 01659. Figure 3 shows the entry of an identified peptide with Uniblue A modification as deposited in the PRIDE database. This peptide was assigned with high significance, expressed by an X!Tandem E-value of 0.0056 and a PeptideProphet probability score of 0.9979.

Trypsin requires a basic arginine or lysine side chain for substrate recognition [26]. Even small chemical modifications that remove the charge, such as methylation or acetic anhydride acetylation result in inhibition of the trypsin activity [27], [28]. Consequently, at positions with Uniblue A derivatized lysines no proteolytic cleavage could be found.

**Figure 4:**
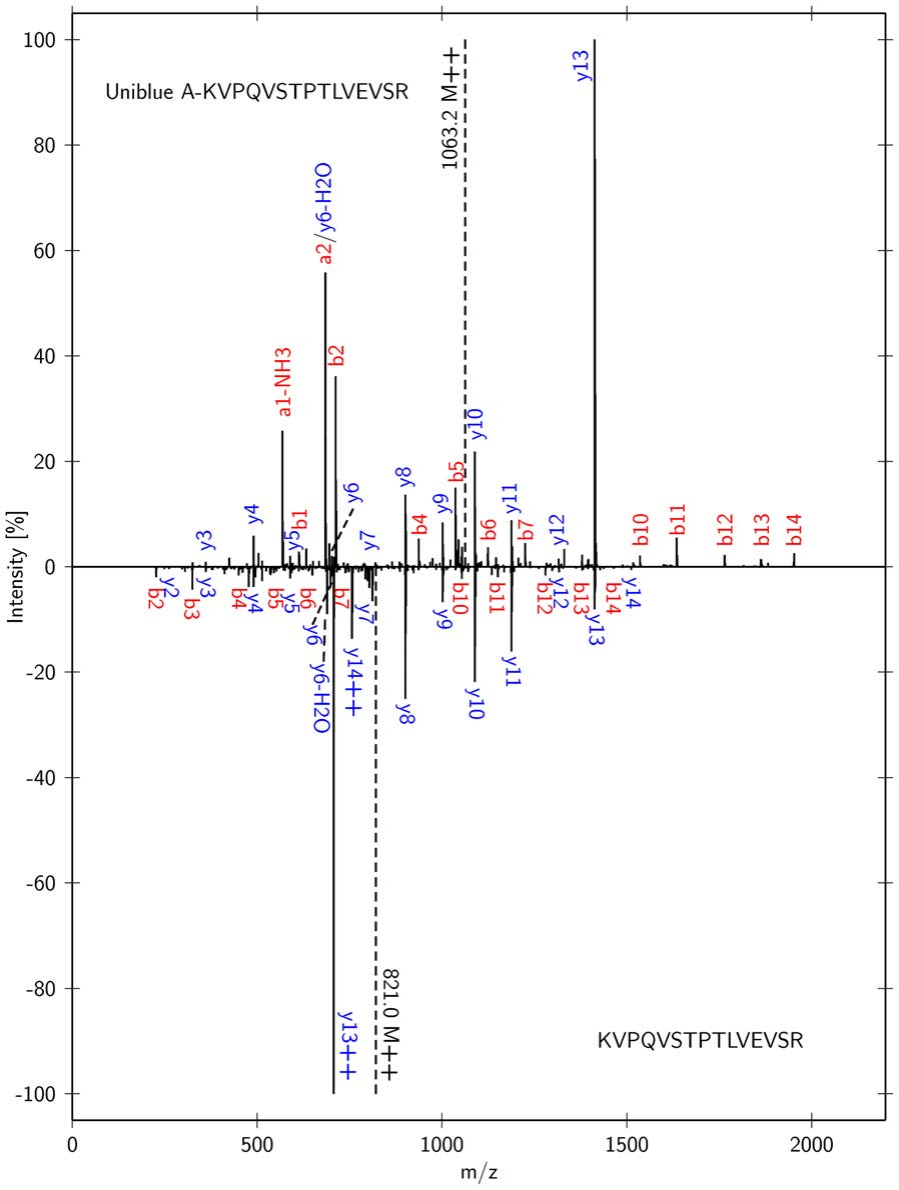
MS/MS fragmentation of an Uniblue A derivatized peptide. The direct comparison of MS/MS fragmentation spectra of Uniblue A derivatized (top) versus un-labeled (bottom) peptide KVPQVSTPTLVEVSR displays significantly increased signal intensities for the derivatized N-terminal ions (a- and b- series, shown in red). The defined mass shift of 484.0399 *m/z* for modified residues allows the detection of fragments, which otherwise would be outside the measuring range (fragments a_1_-NH_3_ and b_1_).

During mass spectrometric analysis, tagged and un-tagged peptides exhibited slightly different behavior. In general, the Uniblue A modification has a tendency to reduce the charge state of the molecules in positive ionization mode due to its negative sulfate group. Figure 4 compares the fragmentation spectrum of a doubly charged Uniblue A derivatized peptide with the fragmentation spectrum of a triply charged untagged peptide of the same sequence. Both spectra were found in the same sample (BSA_dry, PRIDE accession #11793, scans 820 and 1547). In this example, the N-terminal lysine of the tryptic peptide is derivatized. The mass shift allows the clear assignment of the N-terminal fragment ions a_1_-NH_3_ and b_1_, which otherwise would be outside the mass range. Whereas the position of the C-terminal y-ions was not affected, all N-terminal a/b-series ions were shifted, which facilitates the assignment of the peaks b_10_ to b_14_. Additionally, the signal-to-noise ratio of N-terminal ions was significantly improved. Altogether more fragment ions can be assigned automatically for the Uniblue A derivatized peptide.

This finding was further investigated by comparing MS/MS spectra of native and Uniblue A derivatized peptides in the same sample, to exclude other influences such as different concentrations or run-to-run variability. Three MS/MS spectra pairs are provided as **Supplemental Spectra S1**. Especially for short peptides the derivatized peptide spectra provide more evaluable and assignable signals. The E-value of individual peptide identifications was typically improved by at least one order of magnitude for the derivatized version of the peptide compared to the native one.

### Comparison of protein identification results after Uniblue A staining and Coomassie staining

To examine the overall performance of this rapid covalent derivatization protocol in comparison to the standard Coomassie based strategy, we compared the identification results after PeptideProphet/ProteinProphet validation (Tab. 1). Both methods yield identification results which comply with strict acceptance criteria. All proteins were identified with a ProteinProphet probability of 1.0000. At least 6 unique peptides were proven and the MS/MS based sequence coverage was at least 26% in all procedures.

This is remarkable, since analytically challenging proteins have been employed for this study. Bovine serum albumin undergoes post-translational proteolytical processing and contains 17 disulfide bonds. Further, three phosphorylation sites and a copper binding site are probable [29]. Its high lysine content might support the staining, but increase at the same time the data complexity due to partial cleavage events. The Rituximab mouse-human chimeric antibody on the other side, consists of 2×2 subunits, which are connected by disulfide bonds [30]. Additionally, glycosylations might be present. The successful identification of those real-life samples underlines the practical usability of our method.

The reduced number of identified peptides when using only Uniblue A is probably caused by matrix suppression effects during the mass spectrometry, since the samples are washed for less time compared to the Coomassie protocol. This was confirmed by the analysis of samples which were first derivatized with Uniblue A and after electrophoresis stained with Coomassie. For two of the three samples, the double staining led to a dramatically increased number of validated peptides, whereas in only one case the number remained about the same. This demonstrates that Uniblue A derivatization is in principle compatible with mass spectrometry based protein identification. Optimized protocols that address sample-to-sample variation and matrix suppression might further improve possible sequence coverage results.

### Application of Uniblue A derivatization method to complex samples

**Figure 5:**
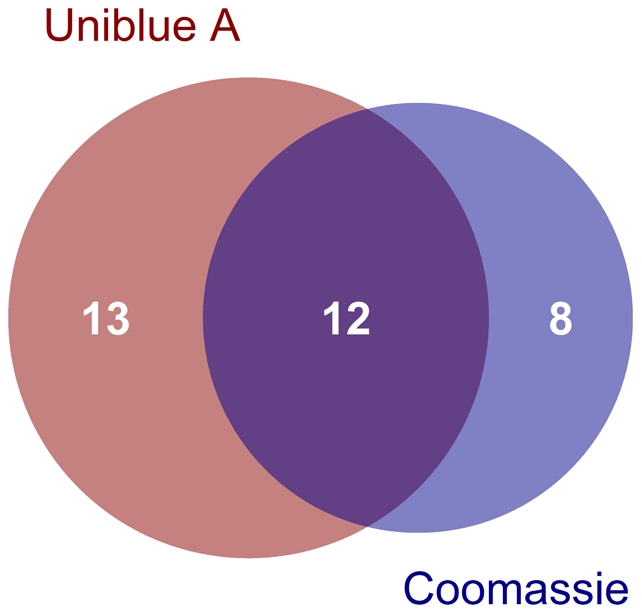
Comparison of identified proteins in the putative *E. coli* MalE-lacZα band. Different proteins were identified from the Uniblue A and the Coomassie stained band of the over-expressed protein. Applying a ProteinProphet score of 0.9 as threshold, 12 proteins were found with both staining methods. 13 proteins were exclusively detected with the Uniblue A staining method, 8 proteins only with the Coomassie staining.

To prove the suitability of the method for complex samples, we applied the covalent derivatization to disintegrates of *Escherichia coli* cells producing a recombinant protein. Uniblue A derivatized and Coomassie stained samples exhibit the sample protein profile (see Fig. 2C), underlining the suitability of the method e.g. for expression clone screening. The supposed recombinant protein at approximately 50 kDa (theoretical molecular weight from sequence: 50,871 Da) was cut and subjected to NanoLC-MS/MS, yielding an excellent MS/MS based sequence coverage above 80% in both cases. Since for some parts of the sequence the data are complementary, the combined MS/MS sequence coverage reaches 92.0% (Fig. S5). Further, the overall detection of proteins in the putative MalE-lacZα bands was compared, to investigate the sensitivity for low abundance proteins (Fig. 5). Applying a ProteinProphet threshold of 0.9, 12 proteins were identified with both staining strategies. Another 13 proteins were found only in the Uniblue A pre-stained band, compared to 8 proteins, which only were detected in the Coomassie stained band. Altogether it can be concluded that both staining strategies exhibit comparable sensitivity for low abundance proteins, and that the staining methods complement each other.

## Discussion

In our study we present a protocol for the rapid staining of proteins and their subsequent analysis by SDS-PAGE and mass spectrometry. The method is applicable for pure proteins as well as for complex mixtures. The SDS-PAGE based protein profiles of derivatized samples are comparable with those of un-derivatized samples in one-dimensional gel electrophoresis, indicating that the electrophoretic mobility in SDS-PAGE is not significantly affected. This was already demonstrated for other covalent protein dyes, namely dabsyl chloride [17] or Remazol dyes [18]. Uniblue A derivatization is not compatible with two-dimensional gel electrophoresis, since the isoelectric properties of the proteins are compromised. The resolution of Uniblue A derivatized proteins is slightly reduced for the 1D-GE, and the staining is less intense compared to standard Commassie staining protocols. However, for the localization of abundant proteins in a 1D-GE gel for subsequent mass spectrometry analysis, those restrictions are not critical. The rapidity of the protocol allows for the “at-line” analysis of proteins. This can be applied for example in the monitoring of the enzymatic cleavage of recombinant fusion proteins.

The required time for sample work-up prior to mass spectrometric analyses can be reduced drastically. Additionally, the visibility of the modified proteins and peptides enables their direct tracking and facilitates the optimization and validation of protocols.

By allowing for different potential derivatization sites in the database search, we could prove that the nucleophilic addition with Uniblue A reaction was highly selective for lysine residues. Integrating the defined mass shift +484.0399 Da at lysine into proteomics software tools enabled automated data evaluation of data sets generated after Uniblue A derivatization. The EBI RESID database for protein modifications does not contain any modification, which would result in a monoisotopic weight correction of 480 to 490 Da [http://www.ebi.ac.uk/RESID/; RESID-CWeightp#480:490]. Therefore the false annotation of a Uniblue A modification is impossible.

Previous studies have demonstrated that sulfonation at the N-term of peptides supports the generation of fragmentation spectra with improved y-series and therefore facilitates peptide identification and *de-novo* sequencing by MALDI-PSD-MS and ESI-MS [31], [32]. The same phenomenon can be observed after derivatization of peptides with Uniblue A, which also contains a sulfone side group, although the derivatization could take place at various positions of a peptide, depending on the location of the lysine. Consequently, Uniblue A tagging is beneficial for automatic or manual peptide identification.

The inhibition of tryptic cleavage at derivatized lysines can be employed to generate longer tryptic peptide fragments. Some peptides, such as YENGKYDIK and KFEKDTGIK in PRIDE Experiment #13516 (MalE-lacZα) only were identified as Uniblue A derivatized peptide. Covalent lysine derivatization by using propionic anhydride, resulting in the neutralization of the charge and thus blocking trypsin cleavages, is employed in the study of histone modifications [33]. Combining tryptic digestion MS/MS data sets of Coomassie stained and Uniblue A derivatized samples improved the total MS/MS based sequence coverage of a recombinant protein. Further, different proteins of lower abundance could be detected. Both findings indicate that those two different staining methods are complementary.

The “peptidic diversity” of tryptic digestions of Uniblue A derivatized proteins is increased by partial derivatization, inhibition of derivatized lysine cleavage sites, different chromatographic properties between un-derivatized and labeled peptides, defined mass shifts during mass spectrometry and different ionization behavior. In theory, this reduces on one side the sensitivity for the mass spectrometric analysis, but on the other side supports improved LC-MS based sequence coverages. The sensitivity of current mass spectrometry instrumentation is already extraordinary high and should not represent a bottleneck. From a practical point of view, we did not experience technical problems in the detection and identification of peptides. The employed algorithms deal well with the potential modifications and the identifications were executed by the automated scripts in about 20 minutes using standard computers.

Further, the enhanced solubility of derivatized proteins and peptides might be used for the analysis and recovery of highly hydrophobic proteins.

Our proposed method is primarily thought to provide a novel and rapid interface between gel electrophoresis and mass spectrometry. This should be especially interesting for the pharmaceutical quality control, but also for speeding up the protein analysis in research and development. Further, the covalent staining has a number of implications concerning the biophysical properties of derivatized proteins and peptides, which can be exploited for defined analytical questions.

Additionally, we invite to think about novel strategies in the development of really mass spectrometry compatible stains and provide a suitable chemical strategy.

## Materials and Methods

### Cultivation of *Escherichia coli* TOP10/pMAL-c4x and production of recombinant protein

*E. coli* TOP10/pMAL-c4x (transformed by electroporation) was grown on Overnight Express™ Instant TB Medium (Novagen Inc.). 50 mL of sterile Overnight Express medium containing 50 µg/ml carbenicillin were given into a 100 mL Erlenmeyer flask and inoculated with 100 µL of a 50% glycerol stock solution of recombinant *E. coli* TOP10/pMAL-c4x (stored at −80°C). The cells were grown for 16 hours at 37°C and 250 rpm. The production of the gene product of the vector, a MalE-lacZα fusion protein was auto-induced after consumption of glucose and the following growth on lactose. The cultivation broth was harvested by centrifugation at 4°C. The pellets were stored at −20°C.

### Disintegration of *E. coli* and TCA/acetone precipitation of intracellular proteins

The *E. coli* pellet was re-suspended in 1 mL of 100 mM carbonate buffer (derivatization buffer solution), transferred into a 1.5 mL Eppendorf tube and sonicated for 5 min. The suspension was clarified by centrifugation at 4°C. Subsequently, 0.1 mL of ice-cooled TCA/acetone solution (1 g/mL TCA in acetone) was added to 0.9 mL of supernatant. This mixture was kept at 4°C for 2 h and centrifuged for 10 min in a pre-cooled micro-centrifuge. The supernatant was discarded and the pellet was washed three times with 1 mL of 90% acetone. Excess of acetone was eliminated by drying and the pellet was re-suspended in 100 µL of 100 mM carbonate buffer (derivatization buffer solution).

### Buffer exchange by ultrafiltration: Preparation of Rituximab antibodies

Commercial MabThera® Rituximab (Hoffmann- La Roche, S.A.) was pre-treated by ultrafiltration. 250 µL of Rituximab (500 mg/50 ml) and 250 µL of 100 mM carbonate buffer (derivatization buffer solution) were placed into a 0.5 ml 3,000 MWCO Amicon® Ultra centrifugal filter unit (Millipore™). After 30 min centrifugation at 14,000× *g*, the permeate was discharged and new carbonate buffer was added to the retentate. This procedure was repeated at least five times. Finally, the Rituximab retentate was resuspended in 125 µL of 100 mM carbonate buffer. The final concentration of Rituximab was 20 mg/mL.

### Production and purification of recombinant amaranth cystatin

Recombinant amaranth cystatin (see Supplemental Sequence S1) was produced and purified as previously described [34]. Briefly, *E. coli* M15 (pREP 4) cells transformed with the pQE-2 vector, containing the amaranth cystatin coding sequence, were grown under agitation at 37°C in Super Broth medium, containing 100 µgmL-1 of carbenicillin and 25 µgmL^−1^ of kanamycin, until they reached an OD600 of 0.5. Cystatin expression was induced by the addition of 0.1 mM IPTG and the cells were harvested after 5 h by centrifugation. The amaranth cystatin was purified from cell lysates using an affinity nickel resin column that was previously equilibrated with 50 mM NaH_2_PO_4_ buffer containing 300 mM NaCl and 10 mM imidazole (pH 8.0). The cystatin was eluted by 250 mM imidazole, dissolved in the same buffer. The purified cystatin was exhaustively dialyzed against water in a microdialysis system (BRL Life technologies, Inc.) with a molecular weight cut-off of 1,000 Da and concentrated in a Savant speedVac vacuum. The protein concentration of the purified cystatin was determined by the BioRad microassay, using serum albumin as standard. Theoretical properties of the recombinant cystatin were calculated using ProtParam [35].

### Uniblue A stain for visualization of proteins

10 µL of 200 mM Uniblue A (Sigma-Aldrich, #298409) solution in derivatization buffer, consisting of 100 mM NaHCO_3_ and 10% SDS, pH 8–9, were added to 90 µL protein solution. Following the sample were heated at 100°C for one minute to perform the staining. Subsequently 100 µL of reducing solution composed of 10% glycerol and 20 mM dithiotreitol (DTT) in 200 mM Tris buffer with pH 6.8, were added in order to reduce cysteins and to adjust the pH for the electrophoresis. Excess Uniblue A reacts with Tris, resulting in a blue compound, which serves as running front indicator in electrophoresis. The sample was heated another minute at 100°C in order to achieve efficient reduction and allowed to cool to room temperature. Subsequently, 20 µL alkylation solution containing 550 mM iodoacetamide (IAA) was added. After 5 minutes incubation time the samples were subjected to SDS-PAGE.

Dry protein samples or samples in compatible buffers (i.e. free of amines) can be diluted directly with the derivatization buffer solution to a protein concentration of 5 mg/mL. In other cases, a prior trichloroacetic acid (TCA)/acetone precipitation or buffer exchange by ultrafiltration is recommended (see below).

As positive control, bovine serum albumin (BSA) in derivatization buffer was used at a concentration of 10 mg/mL.

### 1D-GE, SDS-PAGE

SDS-PAGE was carried out according to the methods of Laemmli [6] and Sambrook [36]. Different concentrations of cystatin and the Uniblue A stained cystatin (10–0.1 µg) were analyzed in SDS–PAGE 12.5%. The protein in the gel was fixed in 40% (v/v) ethanol and 10% (v/v) acetic acid for 20 min and stained with PhastGel Blue R-350 (Amersham, BioScience) following the supplier's instructions. The Uniblue A stained cystatin was detected directly in the gel.

### Two-dimensional (2D) gel electrophoresis

Two-dimensional gel electrophoresis was performed according to the method of Bjellqvist et al. [37]. Dry IPG strips (7 cm long, pH 3–10 linear) were rehydrated at 20 0C for 14 h in 125 µl of isoelectric focusing buffer (7 M urea, 2 M thiourea, 20 mM DTT, 4% CHAPS, 0.5% ampholite 3–10, 0.001% bromophenol blue), containing 1 µg of protein sample. IEF was conducted with an Ettan IPGphor II system (Amersham Biosciences). Focusing was carried out in four steps: 250 V for 1 h, 500 V 0.5 h followed by 1000 V 0.5 h, and finally 8000 V for 2.5 h. After focusing, the gels were equilibrated twice for 15 min in equilibration solution. The first equilibration was performed in a solution containing 6 M urea, 30% w/v glycerol, 2% w/v SDS, 0.001% bromophenol blue, 50 mM Tris-HCl buffer, pH 8.8 and 1% w/v DTT. The second equilibration solution was modified by the replacement of DTT by 2.5% w/v iodoacetamide. For the second dimension, the proteins were separated on 12.5% SDS polyacrylamide gels. Protein spots were visualized using PhastGel Blue R-350 (Amersham Biosciences).

### In-gel digestion for protein bands

For in-gel digestion of protein bands, the protocols of Shevchenko [14], [38] have been adopted with few modifications. In-gel reduction/alkylation is not required since this step is already included in the SDS-PAGE sample preparation.

After the SDS-PAGE, the Uniblue A stained bands could be cut directly from the gel and chopped into cubes with about 1 mm of edge length. The cubes were transferred to vials and covered by acetonitrile. Typically the cubes became whitish and shrunk after about 5 minutes. If not, the acetonitrile solution was exchanged one or two times. The shrunk gel pieces were dried in a vacuum centrifuge. The dry gel pieces were re-hydrated in 10 ng/µL trypsin solution (Promega V511A in 10 mM ammonium bicarbonate) and incubated for 30 min at 60°C. Previous studies had shown that the reductive methylation of Promega trypsin shifts its catalytic optimum to 50–60°C. Therefore, similar peptide yields can be obtained after 30 min digestion at elevated temperatures, compared to overnight digestions at 37°C [39]. After tryptic digestion, the peptides could be extracted by addition of an acetonitrile: 5% trifluoroacetic acid mixture (2∶1) and incubation for 15 min at 60°C.

The extraction solution was collected into a new tube and dried in a vacuum centrifuge. Prior to LC-MS/MS analysis, the peptides were dissolved in 20 µL of 0.1% (v/v) formic acid.

Gels were washed and fixed, if they were going to be scanned or stored. First, the gel was shaken for 5 min in a solution containing 40% methanol and 10% acetic acid, following for 20 min in a solution containing 10% methanol and 7.5% acetic acid. Finally, the gel was washed with the first solution for at least 3 h, until the excess of colorant was eliminated.

### NanoLC-MS/MS measurements

NanoLC-MS/MS analyses were performed on an Agilent 1100 HPLC sytem with nanoLC-ChipCube, coupled with an Agilent LC/MSD Trap XCT Ultra. For instrument control the vendor's programs ChemStation Rev.B.01.03 and TrapControl version 6.1 were used. Solvent A for chromatography was 0.1% formic acid, solvent B 99% acetonitrile in 0.1% formic acid. 8 µL of sample were loaded with a flow of 4 µL/min solvent A on the 40 nL enrichment column of a Agilent G4240-62001 chip column. The flow rate for the analytical chromatography was 0.3 µL/min. After 5 min washing with 3% solvent B, the flow path was changed to the analytical column (43 mm×75 mm, Zorbax 300SB-C18, 5 mm). During the following 27 min the solvent B concentration was increased to 45%. Following the column was cleaned by a 3-minute gradient to 95% solvent B and re-equilibrated 6.5 min with initial conditions. The total time for the chromatography method was 42.5 min. The electron spray ionization was enabled by a capillary voltage of 1,900 V and a nitrogen gas flow of 4 L/min at 325°C. Parent spectra were measured in positive mode, standard-enhanced with an integrated ion current (ICC) smart target setting of 200,000 and a maximal accumulation time of 100 ms. The scan range was from 200 to 1400 m/z. Collision induced dissociation (CID) fragmentation was performed automatically with preference for multiply charged precursor ions. The fragmentation energy was adjusted online by smart parameter setting.

### Evaluation of nanoLC-MS/MS data

In order to enable a target-decoy search strategy [20] with estimation of false positive rates (FDR), a decoy database with reverse sequences was generated and merged with the original one. The forward database was constructed by using the entries of the SwissProt database, supplemented by the sequences of the Rituximab chains, as reported in DrugBank [40] (accession number DB00073), and the theoretical amino acid sequence of the pMAL-c4x vector gene product, MalE-lacZα (sequence derived from technical information of New England BioLabs). The final search data base consisted of approx. 1,000,000 entries.

Agilent XCT ultra *.yep data files were converted to *.mzXML files using the CompassXport 3.0.x program, provided by Bruker. Following the mzXML files were automatically processed by a DOS BATCH script OMSSAVALIDATION.BAT (see below), employing OMSSA 2.1.x [21] as database search engine and PeptideProphet [22] and ProteinProphet [23], versions of Trans-Proteomic Pipeline v4.x [41] for hit validation.

To account for the additional potential protein modification on lysine with Uniblue A, an additional entry was defined in the usermods.xml file in the OMSSA directory:

<MSModSpec>

<MSModSpec_mod>

<MSMod value = “usermod3”>121</MSMod>

</MSModSpec_mod>

<MSModSpec_type>

<MSModType value = “modaa”>0</MSModType>

</MSModSpec_type>

<MSModSpec_name>Uniblue A on K</MSModSpec_name>

<MSModSpec_monomass>484.039891</MSModSpec_monomass>

<MSModSpec_averagemass>484.5016</MSModSpec_averagemass>

<MSModSpec_n15mass>0</MSModSpec_n15mass>

<MSModSpec_residues>

<MSModSpec_residues_E>K</MSModSpec_residues_E>

</MSModSpec_residues>

</MSModSpec>

The modification 121 now is known by OMSSA as “Uniblue A on K” and identified by a mass shift of 484.039891 Da monoisotopic mass, or 484.5016 Da average mass, respectively.

For OMSSA searches, two missed cleavages were allowed, “carbamidomethyl on cysteine” was defined as fixed modification and “deamidation on glutamine/asparagine” and “Uniblue A on lysine” as variable modifications. “Oxidation on methionine” could be omitted, since no methionine oxidation was found in exploratory data base searches. This finding can be attributed to the rapid sample work-up. To allow for subsequent validation of hits by PeptideProphet/ProteinProphet, the e-value was set to 1E6. Precursor and fragment mass tolerances were left at the OMSSA default values, i.e. 2.0 Da for the precursor and 0.8 Da for the fragment masses.

For the PeptideProphet/ProteinProphet hit validations, a minimal peptide length of 5 amino acid residues was specified and a non-parametric validation model based on decoy results.

Raw data and identification results were converted into standard-compliant PRIDE XML using the PRIDE converter, v2.x [25], applying a ProteinProphet probability cut-off of 0.9 and a cut-off for peptides of 0.05. This PRIDE XML files were submitted to the PRIDE server (http://www.ebi.ac.uk/pride/), where the data can be found in the project “Rapid pre-gel visualization of proteins with mass spectrometry compatibility”. Covalent derivatization with Uniblue A has been added by the PRIDE team as a protein modification (PSI-MOD) for the ontology lookup service (OLS) with the comma separated value (CSV) term MOD: 01659.

Manual calculations of fragmentation spectra were conducted with mmass 3.7 [42].

### DOS BATCH script for automated processing of mzXML files

For automated database search and hit validation a DOS BATCH script called OMSSAVALIDATION.BAT has been written. Provided, that the necessary software is installed, the following code can be pasted into a *.bat file and executed on all *.mzXML files of a directory.

@ECHO OFF

ECHO *** Converting mzXML files to mgf ***

FOR %%I IN (*.mzXML) DO MzXML2Search -mgf -ACID -M2-2 -P5 -I0.01 -B10 -T5000.0%%I

ECHO *** OMSSA search: Decoy database, trypsin, pep.xml output ***

REM decoy entries in database customdb start with decoy_; reverse decoy database generated with FastaTools 0.9 and merged with original database using mergeFasta.pl

FOR %%I IN (*.mgf) DO omssacl -d c:\blastdb\customdb -fm %%I -v 2 -mf 3 -mv 4,121 -he 1e6 -w -op %%I_OMSSA_TPP.pep.xml

ECHO *** Fix linking of MS/MS spectra ***

sed -i “s/\.mgf_OMSSA_TPP\.pep\.xml//ig” *OMSSA_TPP.pep.xml

ECHO *** Validation of OMSSA results with PeptideProphet/ProteinProphet; NOOCAM NOGROUPS ***

FOR %%I IN (*OMSSA_TPP.pep.xml) DO interactparser ppval_%%I %%I -L5 -Etrypsin -C -P

FOR %%I IN (ppval*.pep.xml) DO peptideprophetparser %%I DECOY = decoy MINPROB = 0 NONPARAM

FOR %%I IN (ppval*.pep.xml) DO refreshparser %%I c:\blastdb\customdb

FOR %%I IN (ppval*.pep.xml) DO proteinprophet %%I %%I.prot.xml NOOCCAM NOGROUPS

DEL ppval_ppval*

DEL sed*

ECHO *** Standard OMSSA Search ***

FOR %%I IN (*.mgf) DO omssacl -d c:\blastdb\customdb -fm %%I -v 2 -mf 3 -mv 4,121 -w -ob %%I_OMSSA_DECOY.oms

### UV/VIS spectrum of Uniblue A

An aqueous solution of 0.01 mg/mL Uniblue A was measured in a range from 300 to 800 nm against water as blank on a BECKMAN DU 640 spectrometer. The resulting data were converted into ASCII and visualized using a LaTeX typesetting system.

### Venn diagram

The R package VennDiagram [43] was employed for drawing the Venn diagram with R 2.14.0. Proteins of the PRIDE experiments #13515 and #13516 from the analysis of the putative MalE-lacZα fusion protein were included, with a ProteinProphet score of >0.9 as treshold.

### Patent Application

For the pre-gel staining strategy with Uniblue A a patent was filed with the number MX/a/2009/013417.

### Mass spectrometry data availability

Mass spectrometry raw data and identification results have been deposited at the EBI PRIDE server (http://www.ebi.ac.uk/pride/). The data will be made public after acceptance of the manuscript. For review, the data can be accessed with Username: review00783 and Password: JT-FskaG. PRIDE accession codes for individual samples are given in Table 1 or within the manuscript. Peptides modified with Uniblue A are annotated in the individual protein hit view as follows: MOD: 01659 from database MOD at position XX. Monoisotopic Δ: 484.039893, Uniblue A derivatized residue. An example is given in Figure 3.

**Table 1:** NanoLC-MS/MS identification results for gel bands of proteins.

	Bovine serum albumin			Rituximab, heavy chain			Rituximab, light chain			lacZ-á from E.coli	
	Uni A	Uni A+	Coom	Uni A	Uni A+	Coom	Uni A	Uni A+	Coom	Uni A	Coom
		Coom			Coom			Coom			
**MS/MS spectra**	2898	2851	2778	2905	2818	2852	2887	2818	2869	2980	2838
**ProteinProphet Probability**	1	1	1	1	1	1	1	1	1	1	1
**MS/MS Sequence coverage**	32.5%	52.1%	40.2%	27.7%	26.0%	41.6%	33.8%	58.1%	62.9%	82.3%	83.8%
**Total ident. peptides**	43	90	93	19	14	102	20	55	125	111	120
**Uniblue A deriv.**	0	17	N.A.	0	0	N.A.	0	0	N.A.	6	N.A.
**PRIDE accession #**	12567	12565	12564	12571	12569	12568	12575	12573	12572	13516	13515

Proteins were either covalently labeled with Uniblue A (Uni A) before electrophoresis or stained with Coomassie (Coom) after electrophoresis. Also sequential staining with both methods was applied (Uni A+Coom). The bands of interest were cut, tryptically digested and subjected to nanoLC-MS/MS identification (see Methods).

## Supporting Information

Sequence S1 **Recombinant amaranth cystatin fasta sequence.**
(DOC)

Figure S1 **UV/VIS spectrum of Uniblue A (0.01 mg/mL in water).** Uniblue A exhibits strong absorption in the visible wavelength region with a maximum λ_max_ at 593.5 nm and a shoulder at about 630 nm.
(DOC)

Figure S2 **Comparison of electrophoretic mobility of Uniblue A derivatized recombinant cystatin with the electrophoretic mobility of un-derivatized cystatin (Coomassie staining).** There is no significant change of the electrophoretic mobility of cystatin detectable.
(DOC)

Figure S3 **Evaluation of the sensitivity of Uniblue A pre-staining using recombinant cystatin.** Gel A shows the gel directly after running and fixation, gel B after additional staining with PhastGel Blue R. Uniblue A derivatized cystatin bands can be detected visually down to about 1 µg of loaded protein. Subsequent staining with PhastGel Blue R allows the detection of about 0.1 µg of loaded protein.
(DOC)

Figure S4 **Two-dimensional gel electrophoresis of Uniblue A derivatized recombinant cystatin.** Uniblue A derivatized recombinant cystatin cannot be focused, even after 18930Vh of isoelectric-focusing. An increasing derivatization degree leads to a shift of the isoelectric point of the protein towards the basic region. Further, the band gets more diffuse, although the apparent molecular weight does not change significantly.
(DOC)

Figure S5 **MS/MS based sequence coverage for MalE-lacZα fusion protein from** ***E. coli*** **expression vector pMAL-c4x.**
(DOC)

Spectra S1 Supplemental MS/MS spectra of native and Uniblue A derivatized peptides.
(DOC)
