# Supplementary material for: Accelerated Identification of Proteins by Mass Spectrometry by Employing Covalent Pre-Gel Staining with Uniblue A
Source: PLoS One. 2012 Feb 17;7(2):e31438. doi: 10.1371/journal.pone.0031438 (PMC3281962; doi:10.1371/journal.pone.0031438)
Supplement: Sequence S1 — Recombinant amaranth cystatin fasta sequence. (DOC) [file pone.0031438.s001.doc]

**Sequence S1.** Recombinant amaranth cystatin fasta sequence.

>Cystatin, Amaranthus hypochondriacus, recombinant
MKHHHHHHHQ ATLGGLRESQ GAANDAEIES LARFAVDEHN KKENALLEFA RVVKAKEQVV AGTLHHFTIE AIDAGKKKLY DAKVWVKPWM NFKELQEFKH TEDSPSFTSS DLGAIREGHA PGWKEVPVHD PEVQNAAEHA VKTIQQRSNS LFPYELQEIA HAKAEVVEDT AKFNLHLKVK RGNKDEIFNV EVHKSSDGNY NLNKMGNIQP EIENQ

Characteristics for recombinant cystatin, as determined by ProtParam:

Number of amino acids: 215;

Molecular weight: 24,382.2 Da; Theoretical pI: 6.27;

0 Cysteins; 21 Lysines
