# Supplementary material for: Accelerated Identification of Proteins by Mass Spectrometry by Employing Covalent Pre-Gel Staining with Uniblue A
Source: PLoS One. 2012 Feb 17;7(2):e31438. doi: 10.1371/journal.pone.0031438 (PMC3281962; doi:10.1371/journal.pone.0031438)
Supplement: Figure S1 — UV/VIS spectrum of Uniblue A (0.01 mg/mL in water). Uniblue A exhibits strong absorption in the visible wavelength region with a maximum λmax at 593.5 nm and a shoulder at about 630 nm. (DOC) [file pone.0031438.s002.doc]

**Figure S1.** UV/VIS spectrum of Uniblue A (0.01 mg/mL in water).


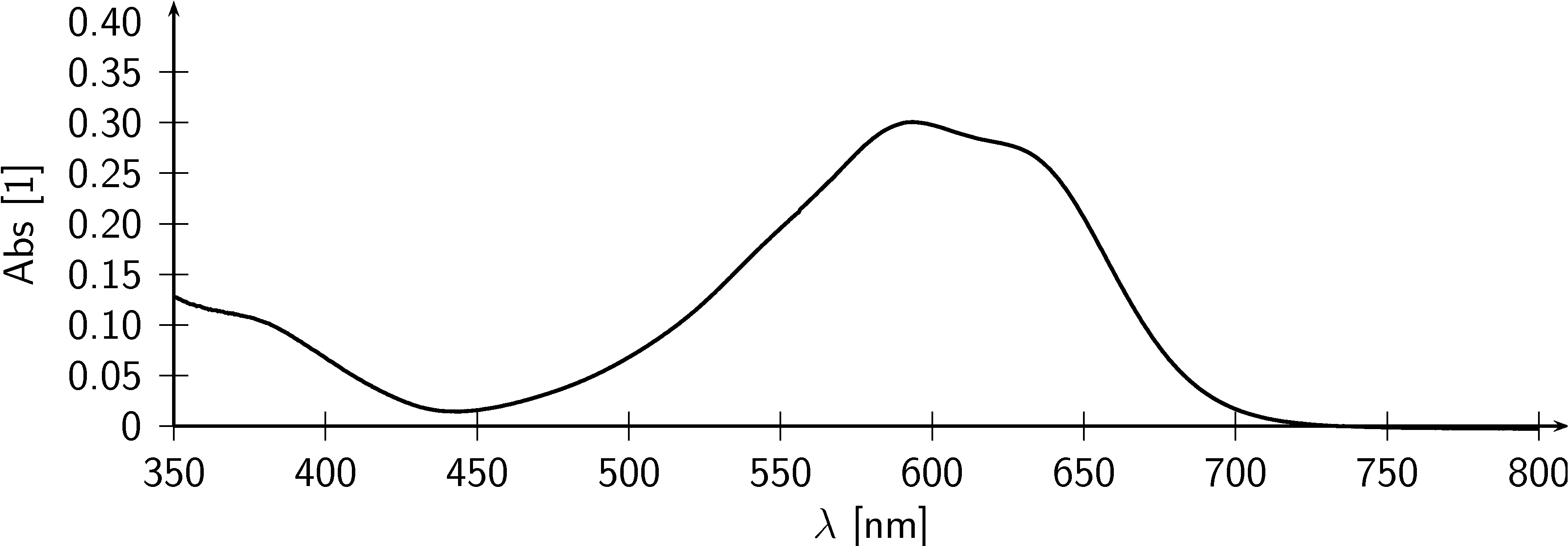


Uniblue A exhibits strong absorption in the visible wavelength region with a maximum λ_max_ at 593.5 nm and a shoulder at about 630 nm.
