# Supplementary material for: Accelerated Identification of Proteins by Mass Spectrometry by Employing Covalent Pre-Gel Staining with Uniblue A
Source: PLoS One. 2012 Feb 17;7(2):e31438. doi: 10.1371/journal.pone.0031438 (PMC3281962; doi:10.1371/journal.pone.0031438)
Supplement: Figure S2 — Comparison of electrophoretic mobility of Uniblue A derivatized recombinant cystatin with the electrophoretic mobility of un-derivatized cystatin (Coomassie staining). There is no significant change of the electrophoretic mobility of cystatin detectable. (DOC) [file pone.0031438.s003.doc]

**Figure S2.** Comparison of electrophoretic mobility of Uniblue A derivatized recombinant cystatin with the electrophoretic mobility of un-derivatized cystatin (Coomassie staining).

**
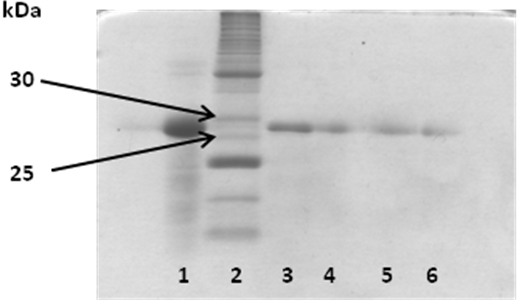
**

1 - cystatin Uniblue A derivatized, 5 µg;

2 - molecular weight marker;

3 - cystatin un-derivatized, 0.5 µg;

4 to 6 - Uniblue A derivatized, 0.5 µg.

There is no significant change of the electrophoretic mobility of cystatin detectable.
