# Supplementary material for: Accelerated Identification of Proteins by Mass Spectrometry by Employing Covalent Pre-Gel Staining with Uniblue A
Source: PLoS One. 2012 Feb 17;7(2):e31438. doi: 10.1371/journal.pone.0031438 (PMC3281962; doi:10.1371/journal.pone.0031438)
Supplement: Figure S3 — Evaluation of the sensitivity of Uniblue A pre-staining using recombinant cystatin. Gel A shows the gel directly after running and fixation, gel B after additional staining with PhastGel Blue R. Uniblue A derivatized cystatin bands can be detected visually down to about 1 µg of loaded protein. Subsequent staining with PhastGel Blue R allows the detection of about 0.1 µg of loaded protein. (DOC) [file pone.0031438.s004.doc]

**Figure S3.** Evaluation of the sensitivity of Uniblue A pre-staining using recombinant cystatin. Gel A shows the gel directly after running and fixation, gel B after additional staining with PhastGel Blue R.

| **A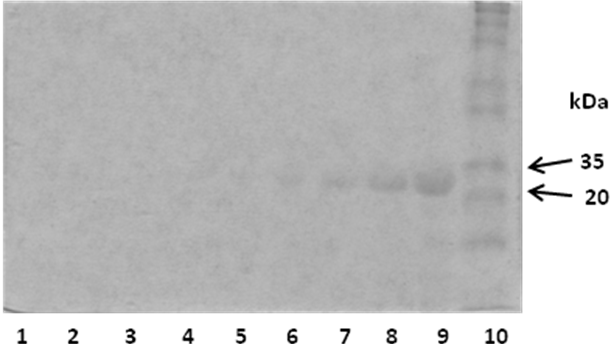B** | **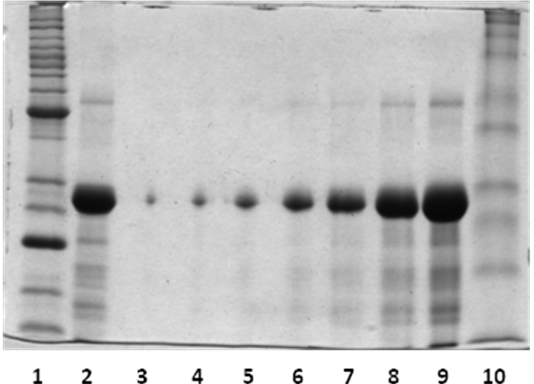** |
| --- | --- |

1 - molecular weight marker, unstained; 2 - cystatin un-derivatized, 5 µg; 3 – cystatin Uniblue A derivatized, 0.1 µg; 4 – cystatin Uniblue A derivatized, 0.2 µg; 5 – cystatin Uniblue A derivatized, 0.5 µg; 6 – cystatin Uniblue A derivatized, 1 µg; 7 – cystatin Uniblue A derivatized, 2 µg; 8 – cystatin Uniblue A derivatized, 5 µg; 9 – cystatin Uniblue A derivatized, 10 µg; 10 - molecular weight marker, pre-stained.

Uniblue A derivatized cystatin bands can be detected visually down to about 1 µg of loaded protein. Subsequent staining with PhastGel Blue R allows the detection of about 0.1 µg of loaded protein.
