# Supplementary material for: Accelerated Identification of Proteins by Mass Spectrometry by Employing Covalent Pre-Gel Staining with Uniblue A
Source: PLoS One. 2012 Feb 17;7(2):e31438. doi: 10.1371/journal.pone.0031438 (PMC3281962; doi:10.1371/journal.pone.0031438)
Supplement: Figure S4 — Two-dimensional gel electrophoresis of Uniblue A derivatized recombinant cystatin. Uniblue A derivatized recombinant cystatin cannot be focused, even after 18930Vh of isoelectric-focusing. An increasing derivatization degree leads to a shift of the isoelectric point of the protein towards the basic region. Further, the band gets more diffuse, although the apparent molecular weight does not change significantly. (DOC) [file pone.0031438.s005.doc]

**Figure S4.** Two-dimensional gel electrophoresis of Uniblue A derivatized recombinant cystatin.

**
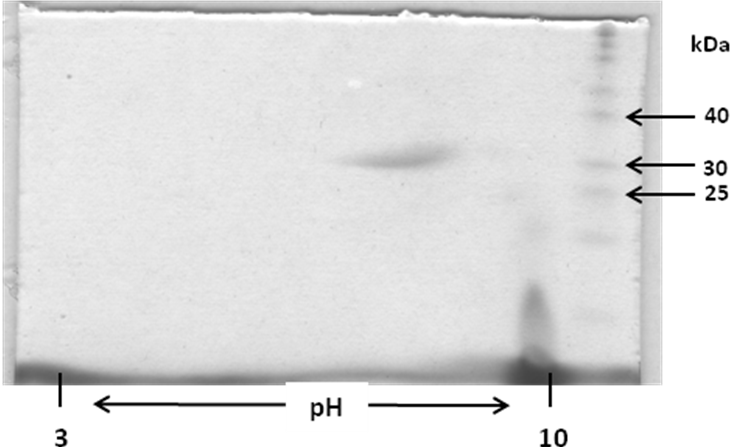
**

Uniblue A derivatized recombinant cystatin cannot be focused, even after 18930Vh of isoelectric-focusing. An increasing derivatization degree leads to a shift of the isoelectric point of the protein towards the basic region. Further, the band gets more diffuse, although the apparent molecular weight does not change significantly.
