# Supplementary material for: Accelerated Identification of Proteins by Mass Spectrometry by Employing Covalent Pre-Gel Staining with Uniblue A
Source: PLoS One. 2012 Feb 17;7(2):e31438. doi: 10.1371/journal.pone.0031438 (PMC3281962; doi:10.1371/journal.pone.0031438)
Supplement: Figure S5 — MS/MS based sequence coverage for MalE-lacZα fusion protein from E. coli expression vector pMAL-c4x. (DOC) [file pone.0031438.s006.doc]

**Figure S5.** MS/MS based sequence coverage for MalE-lacZα fusion protein from *E. coli* expression vector pMAL-c4x.

1. MS/MS based sequence coverage after covalent modification with Uniblue A
   380/462 amino acids = 82.2%

0001  MKIEEGKLVI WINGDKGYNG LAEVGKKFEK DTGIKVTVEH PDKLEEKFPQ VAATGDGPDI IFWAHDRFGG YAQSGLLAEI  0080
0081  TPDKAFQDKL YPFTWDAVRY NGKLIAYPIA VEALSLIYNK DLLPNPPKTW EEIPALDKEL KAKGKSALMF NLQEPYFTWP  0160
0161  LIAADGGYAF KYENGKYDIK DVGVDNAGAK AGLTFLVDLI KNKHMNADTD YSIAEAAFNK GETAMTINGP WAWSNIDTSK  0240
0241  VNYGVTVLPT FKGQPSKPFV GVLSAGINAA SPNKELAKEF LENYLLTDEG LEAVNKDKPL GAVALKSYEE ELVKDPRIAA  0320
0321  TMENAQKGEI MPNIPQMSAF WYAVRTAVIN AASGRQTVDE ALKDAQTNSS SNNNNNNNNN NLGIEGRISE FGSSRVDLQA  0400
0401  SLALAVVLQR RDWENPGVTQ LNRLAAHPPF ASWRNSEEAR TDRPSQQLRS LNGEWQLGCF GG

1. MS/MS based sequence coverage after Coomassie staining 387/462 amino acids = 83.8%

0001  MKIEEGKLVI WINGDKGYNG LAEVGKKFEK DTGIKVTVEH PDKLEEKFPQ VAATGDGPDI IFWAHDRFGG YAQSGLLAEI  0080
0081  TPDKAFQDKL YPFTWDAVRY NGKLIAYPIA VEALSLIYNK DLLPNPPKTW EEIPALDKEL KAKGKSALMF NLQEPYFTWP  0160
0161  LIAADGGYAF KYENGKYDIK DVGVDNAGAK AGLTFLVDLI KNKHMNADTD YSIAEAAFNK GETAMTINGP WAWSNIDTSK  0240
0241  VNYGVTVLPT FKGQPSKPFV GVLSAGINAA SPNKELAKEF LENYLLTDEG LEAVNKDKPL GAVALKSYEE ELVKDPRIAA  0320
0321  TMENAQKGEI MPNIPQMSAF WYAVRTAVIN AASGRQTVDE ALKDAQTNSS SNNNNNNNNN NLGIEGRISE FGSSRVDLQA  0400
0401  SLALAVVLQR RDWENPGVTQ LNRLAAHPPF ASWRNSEEAR TDRPSQQLRS LNGEWQLGCF GG

1. Combined sequence coverage
   425/462 amino acids = 92.0%

0001  MKIEEGKLVI WINGDKGYNG LAEVGKKFEK DTGIKVTVEH PDKLEEKFPQ VAATGDGPDI IFWAHDRFGG YAQSGLLAEI  0080
0081  TPDKAFQDKL YPFTWDAVRY NGKLIAYPIA VEALSLIYNK DLLPNPPKTW EEIPALDKEL KAKGKSALMF NLQEPYFTWP  0160
0161  LIAADGGYAF KYENGKYDIK DVGVDNAGAK AGLTFLVDLI KNKHMNADTD YSIAEAAFNK GETAMTINGP WAWSNIDTSK  0240
0241  VNYGVTVLPT FKGQPSKPFV GVLSAGINAA SPNKELAKEF LENYLLTDEG LEAVNKDKPL GAVALKSYEE ELVKDPRIAA  0320
0321  TMENAQKGEI MPNIPQMSAF WYAVRTAVIN AASGRQTVDE ALKDAQTNSS SNNNNNNNNN NLGIEGRISE FGSSRVDLQA  0400
0401  SLALAVVLQR RDWENPGVTQ LNRLAAHPPF ASWRNSEEAR TDRPSQQLRS LNGEWQLGCF GG
