# Supplementary material for: Accelerated Identification of Proteins by Mass Spectrometry by Employing Covalent Pre-Gel Staining with Uniblue A
Source: PLoS One. 2012 Feb 17;7(2):e31438. doi: 10.1371/journal.pone.0031438 (PMC3281962; doi:10.1371/journal.pone.0031438)

**Spectra S1.** Supplemental MS/MS spectra of native and Uniblue A derivatized peptides.

**Sample “BSA_dry”, ALKAWSVAR, native**

**E-value: 0.50792**

**
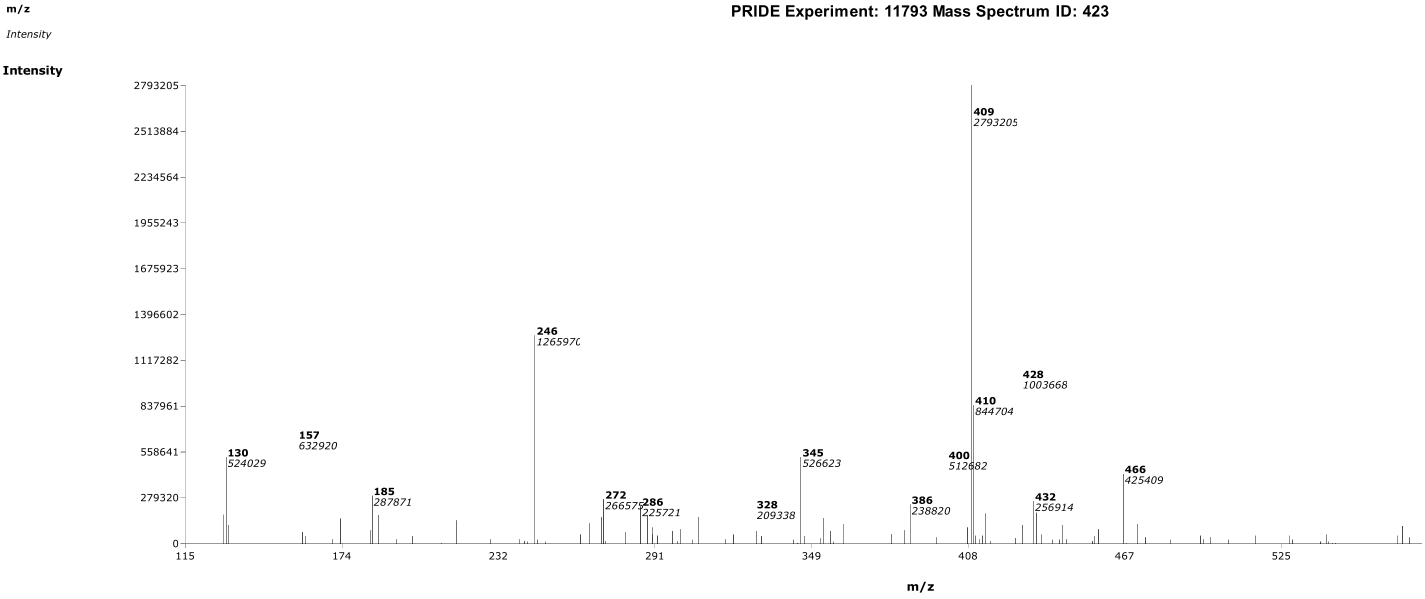
**

**Sample “BSA_dry”, ALKAWSVAR, Uniblue A derivatization at lysine (pos. 3)**

**E-value: 0.03921**


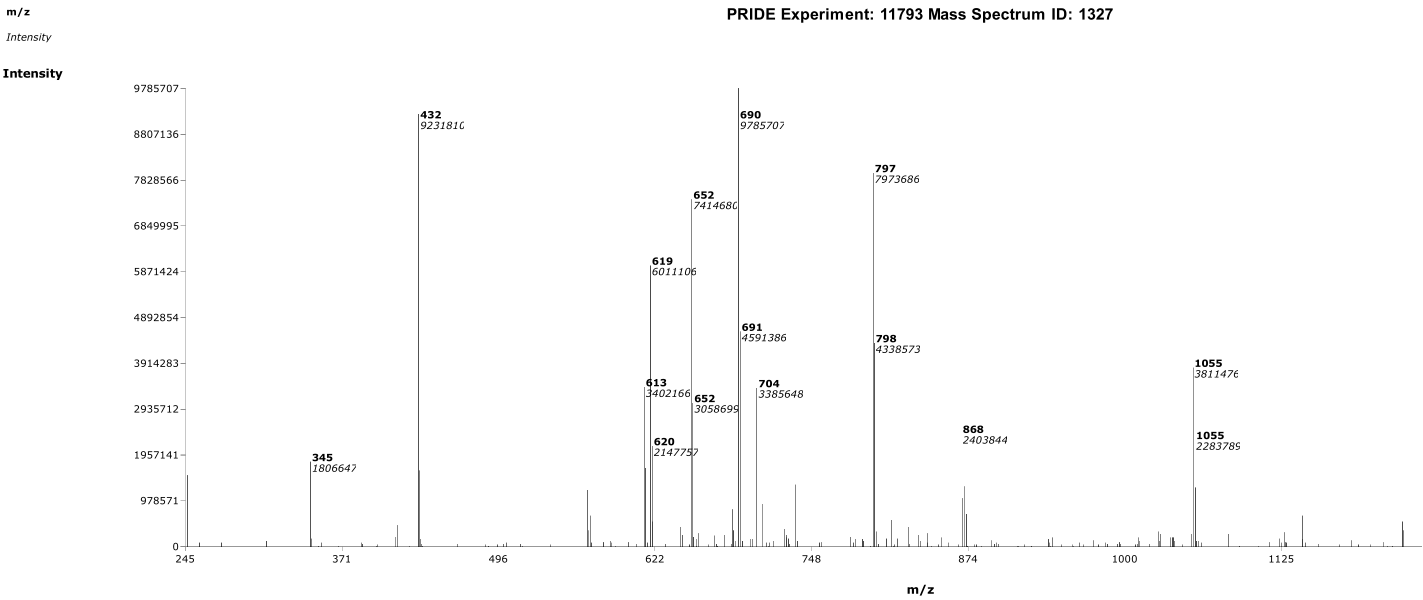


**Sample “pmal-c4x_UniA”, TWEEIPALDKELK, native**

**E-value: 0.09724**

**
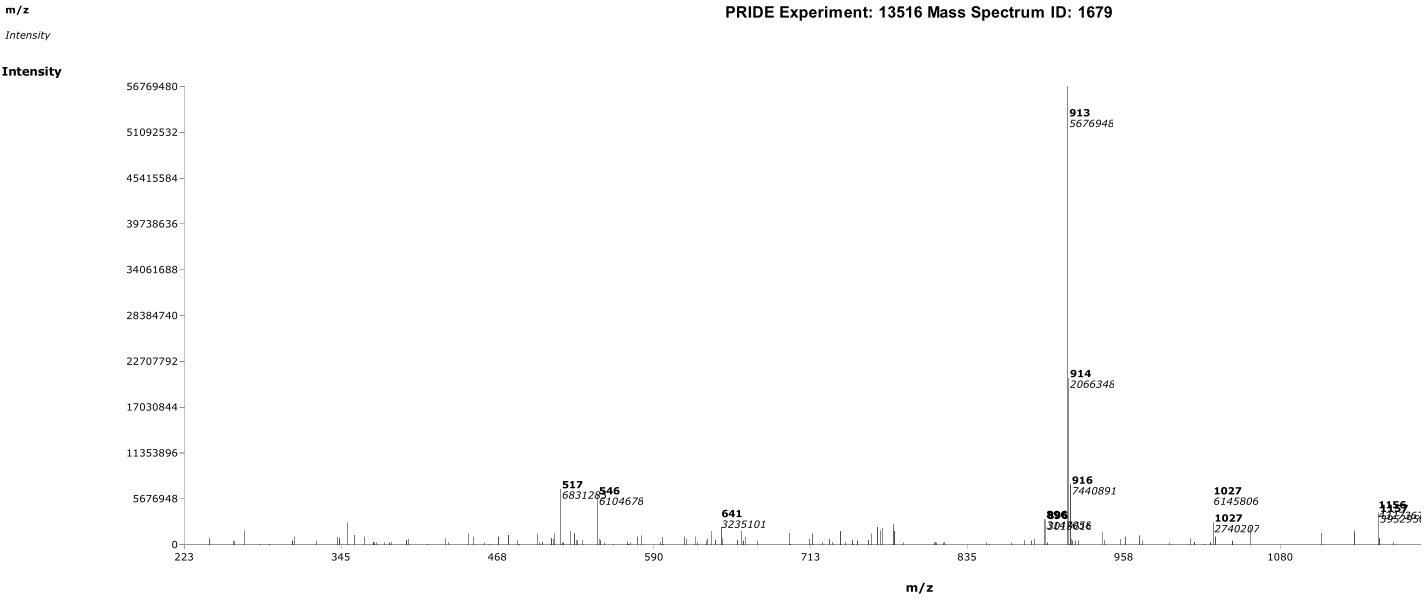
**

**Sample “pmal-c4x_UniA”, TWEEIPALDKELK, Uniblue A derivatization at lysine (pos. 10)**

**E-value: 0.00120**


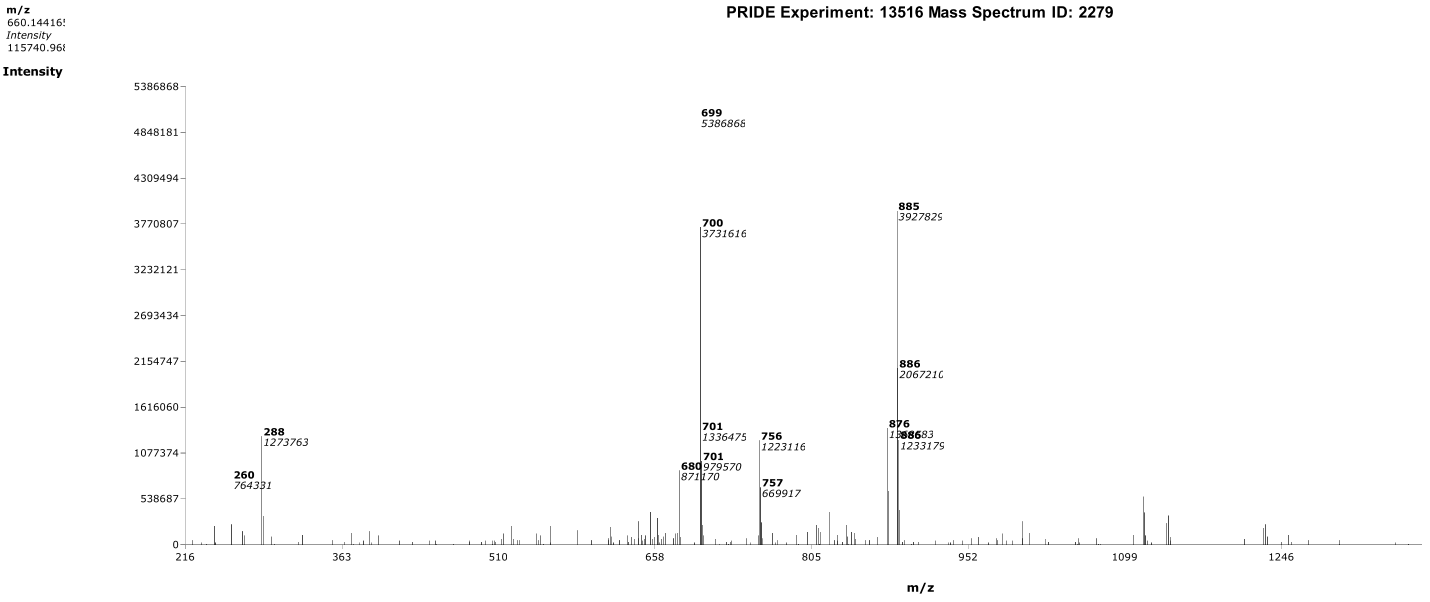


**Sample “BSA_UniA_Coom”, FKDLGEEHFK, native**

**E-value: 177.28837**

**
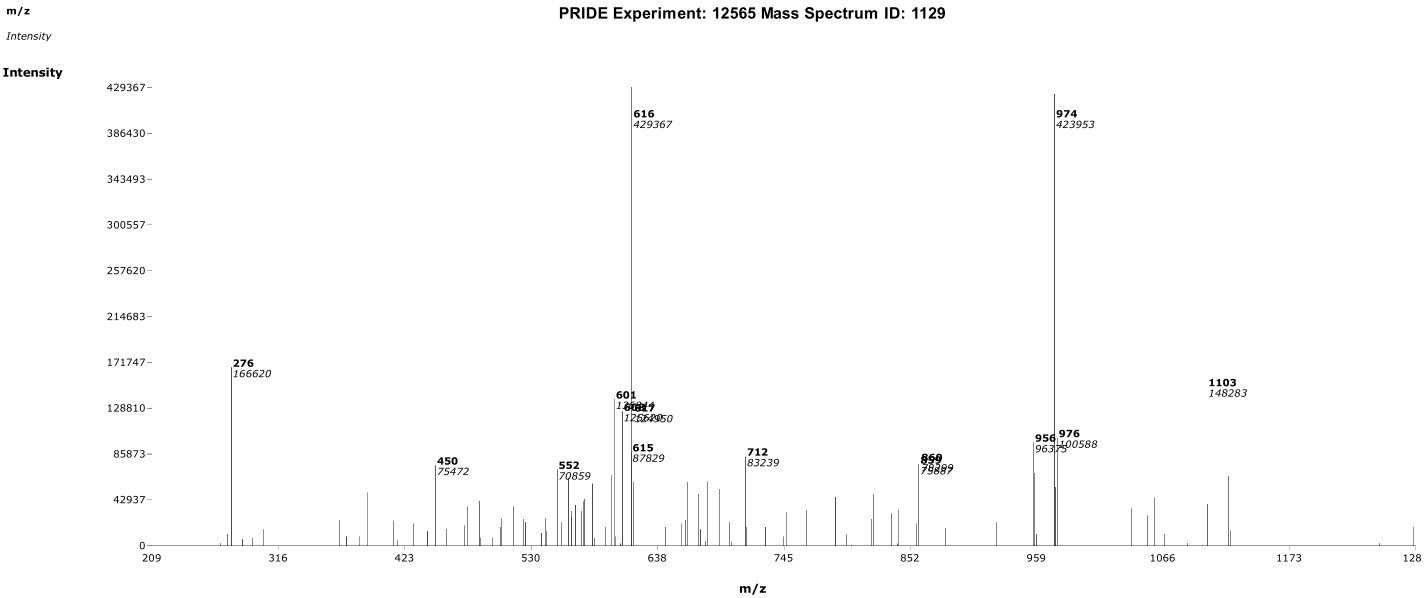
**

**Sample “BSA_UniA_Coom”, FKDLGEEHFK, Uniblue A derivatization at lysine (pos. 2)**

**E-value: 0.00071**


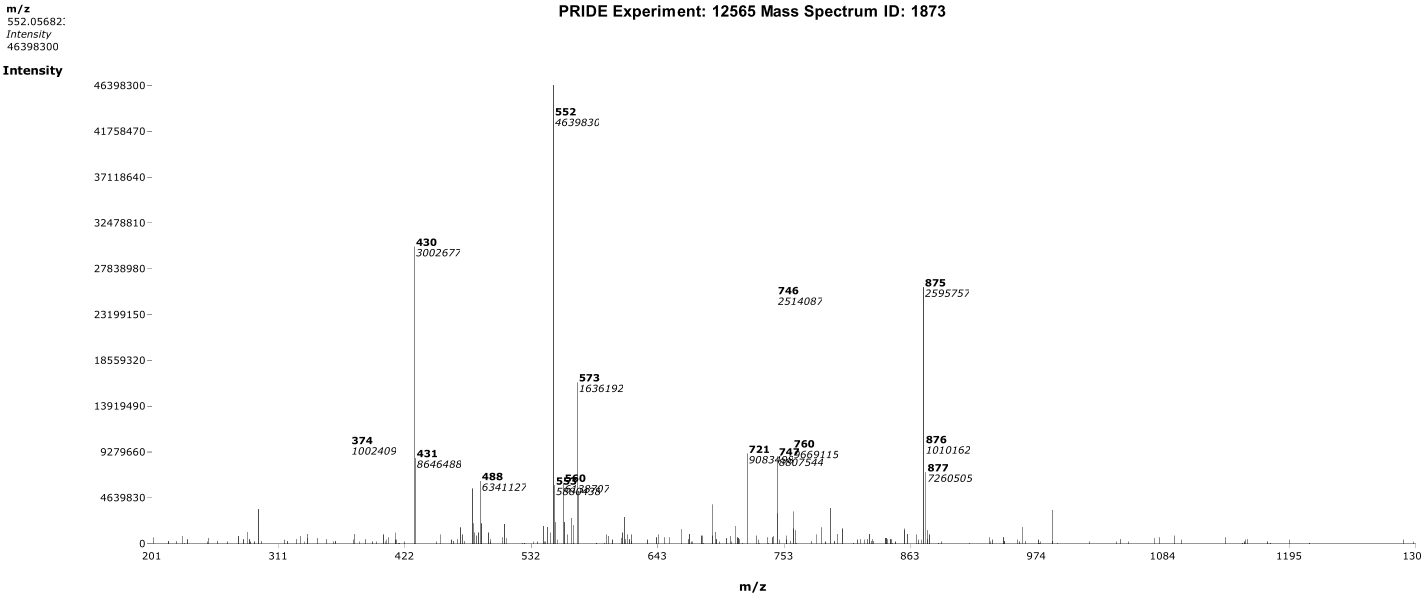

Supplement: Spectra S1 — Supplemental MS/MS spectra of native and Uniblue A derivatized peptides. (DOC) [file pone.0031438.s007.doc]
